# An Atypical Presentation of Tuberculous Ascites, Severe Hyponatremia, and Acute Kidney Injury in a Young Male

**DOI:** 10.1002/ccr3.72230

**Published:** 2026-04-24

**Authors:** Bassem Al Hariri, Muhammad Sharif, Memon Noor Illahi, Asgad Mohamed Ahmed Eltigani, Usamah Saad Mohammad Al Anbagi, Muayad Ahmad

**Affiliations:** ^1^ Department of Medicine Hamad Medical Corporation, HMGH Doha Qatar; ^2^ College of Medicine Qatar University Doha Qatar; ^3^ College of Medicine Weill Cornell Medicine – Qatar Doha Qatar; ^4^ Medical Education Department Hamad Medical Corporation Doha Qatar

**Keywords:** acute kidney injury (AKI), extrapulmonary tuberculosis, hyponatremia, interstitial nephritis, paracentesis, tuberculous ascites

## Abstract

A 25‐year‐old male who had been in good health before presenting with right lower lobe pneumonia, severe hyponatremia (Na 117 mEq/L), distension, and reduced urine output. AKI secondary to acute glomerulonephritis (GN) or interstitial nephritis was suggested by the initial workup. He was discharged for outpatient nephrology follow‐up following initial stabilization with hypertonic saline and intravenous fluids; however, 2 days later, he was readmitted due to worsening fever and ascites. Tuberculous peritonitis was confirmed by paracentesis, which also showed an AFB smear and TB PCR‐positive ascitic fluid. Clinical improvement led to the initiation of antitubercular therapy (ATT). This case emphasizes that TB should be considered in the differential diagnosis of unexplained ascites and AKI even in immunocompetent patients without pulmonary symptoms, and that ascitic fluid PCR is a critical diagnostic tool in endemic regions.

## Key Clinical Message

A 25‐year‐old immunocompetent man presented with ascites, severe hyponatremia, and acute kidney injury. This case highlights that extrapulmonary tuberculosis should be a key diagnostic consideration in unexplained ascites with renal involvement, even without pulmonary symptoms. Early ascitic fluid TB‐PCR analysis is crucial for prompt diagnosis and treatment in endemic regions.

## 1 Introduction

Tuberculous peritonitis, though rare, is a clinically important form of extrapulmonary tuberculosis (EPTB) that frequently manifests with nonspecific symptoms, making prompt diagnosis difficult. Although the most prevalent type is pulmonary tuberculosis, EPTB can impact any organ system, with peritoneal involvement representing about 2% of cases [1]. The challenge of diagnosis is even greater in immunocompetent individuals, as atypical manifestations like ascites, acute kidney injury (AKI), and severe hyponatremia can obscure the true cause, resulting in treatment delays and worse outcomes.

The case report details a previously healthy 25‐year‐old man who presented with a triad of severe hyponatremia (Na 117 mEq/L), AKI, and ascites, initially thought to be due to acute glomerulonephritis or interstitial nephritis. Even in the absence of typical tuberculosis symptoms like fever, night sweats, or weight loss, further examinations showed tuberculous peritonitis, which was confirmed by AFB smear and TB PCR‐positive ascitic fluid. The clinical course of the patient highlights the necessity of keeping a high level of suspicion for EPTB in endemic areas, even when there is no pulmonary involvement or immunocompromised condition.

This report emphasizes the importance of early diagnostic paracentesis and PCR‐based testing for enhancing detection rates [2], as well as the challenges involved in managing concurrent hyponatremia and AKI in tuberculosis cases [3]. Through the investigation of this unusual presentation, we intend to highlight the importance of collaboration across various medical disciplines and the incorporation of sophisticated diagnostic tools in order to enhance patient outcomes in analogous situations. The results also support the addition of TB PCR to routine analyses of ascitic fluid in regions where TB is prevalent, in order to cut down on delays in diagnosis and improve treatment effectiveness [2].

## 2 Case Presentation

A 25‐year‐old male who had been in good health for 2 weeks had been experiencing progressive, nonradiating stomach pain that was accompanied by early satiety, decreased urine output, and growing distension. He did not mention any recent travel or TB contacts. Initially, he denied any history of fever, night sweats, or weight loss. However, upon more detailed questioning during his readmission, he recalled experiencing subjective low‐grade fevers and fatigue intermittently over the month prior to his initial presentation. He still had no known TB contacts and had not traveled recently. Previous Medical and Social History: No history of long‐term conditions, such as diabetes, high blood pressure, or liver disease. No history of immunosuppressive treatment or TB exposure. None smoker, does not use intravenous drugs, and occasionally consumes alcohol.

Physical Examination: Vital Signs: normotensive (BP 125/80 mmHg), febrile (38.5°C), and 98% saturating in room air. Chest: Dullness to percussion and decreased air entry in the lower right lobe, indicating consolidation or diffusion. Abdomen: No discomfort or guarding, but noticeable distension and fluctuating dullness. The sounds of the bowels were normal. Bilateral pitting edema (2+) up to the knees is extreme.

## 3 Initial Diagnostic Suspicion

Given the triad of ascites, AKI, and hyponatremia, the differential diagnosis included:

- *Infectious causes*: Tuberculous peritonitis, bacterial peritonitis.
- *Malignancy*: Peritoneal carcinomatosis (less likely in a young patient).
- *Nephrogenic causes*: Acute interstitial nephritis (drug‐induced vs. infection‐related), glomerulonephritis.
- *Hepatic causes*: Cirrhosis (ruled out by absent stigmata and normal liver function).

## 4 Diagnostic Investigations

### 4.1 Laboratory Findings

Severe hyponatremia (Na 117 mEq/L), AKI (creatinine 146 μmol/L), heavy proteinuria (2.13 g/24 h), and elevated inflammatory markers (CRP 120 mg/L). Normal lipase (78 U/L), no hyperkalemia, or adrenal insufficiency. TB work up in urine was negative (Table 1). PPD or IGRA (T‐SPOT) tests were not performed, as they cannot differentiate between latent and active disease, and a definitive diagnosis was pursued through ascitic fluid analysis.

**TABLE 1:** Laboratory results.

Parameter	Patient's value			Reference range
	Admission	Mid stay	Discharge	
WBC (×10^3^/μL)	8.8	8.2	7.9	4–10
RBC (×10^6^/μL)	4.4	4.4	3.9	4.5–5.5
HGB (g/dL)	11.2	11.5	10.2	13‐17
HCT (%)	34.4	33.1	31.4	40–50
MCV	78.9	76.1	80.5	83–101
MCH	25.7	26.4	26.2	27–32
MCHC	32.6	34.7	32.5	31.5–34.5
RDW‐CV (%)	12.6	12.7	15.9	11.6–14.5
Platelets (×10^3^/μL)	306	334	317	150–400
Absolute neutrophil count auto (×10^3^/μl)	7	6.5	4.7	2–7
Urea (mmol/L)	9.1	12.0	16.1	2.5‐7.8
Creatinine (μmol/L)	146	130	110	62–106
Sodium (mmol/L)	117	121	131	133–146
Chloride (mmol/L)	98	82	100	95–108
CRP (mg/L)	120	54.7	32.4	0.0–5.0
Albumin (g/L)	25	24	33	35–50
U24 total volume	606	600	1653	800–2000
U24 Protein (g/24 h)	2.13		0.08	0.03–0.15
ASO (IU/mL)	480	1446		0–200

Abbreviations: ASO, ant streptolysin O titer; CRP, C‐Reactive protein; HCT, hematocrit; HGB, hemoglobin; MCH, mean corpuscular hemoglobin; MCHC, mean corpuscular hemoglobin concentration; MCV, mean corpuscular volume; RBC, red blood cell count; RDW‐CV, red cell distribution width‐coefficient of variation; WBC, white blood cell count.

### 4.2 Imaging

**FIGURE 1:**
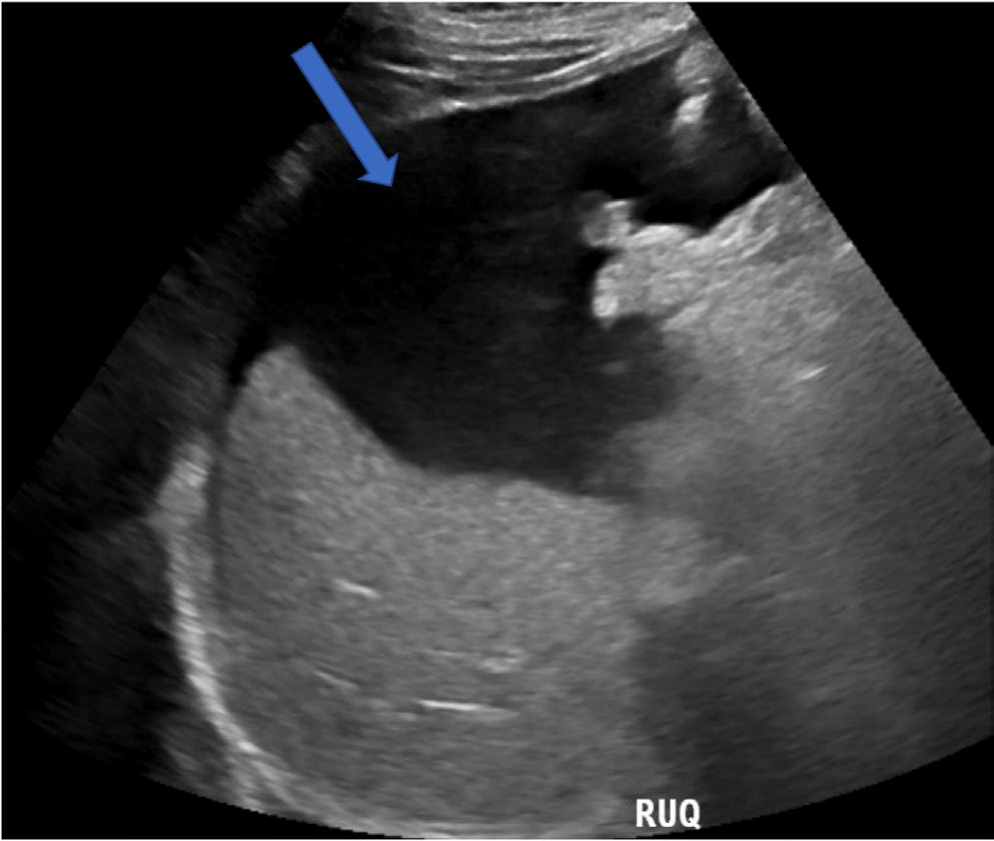
Abdomen ultrasound moderate‐to‐large ascites (blue arrow).

Abdomen ultrasound (04/17/2025): Moderate‐to‐large ascites; kidneys with increased cortical echogenicity but normal size (right: 12 × 5 cm; left: 12 × 5.7 cm) and preserved corticomedullary differentiation. No hydronephrosis or masses (Figure 1).

**FIGURE 2:**
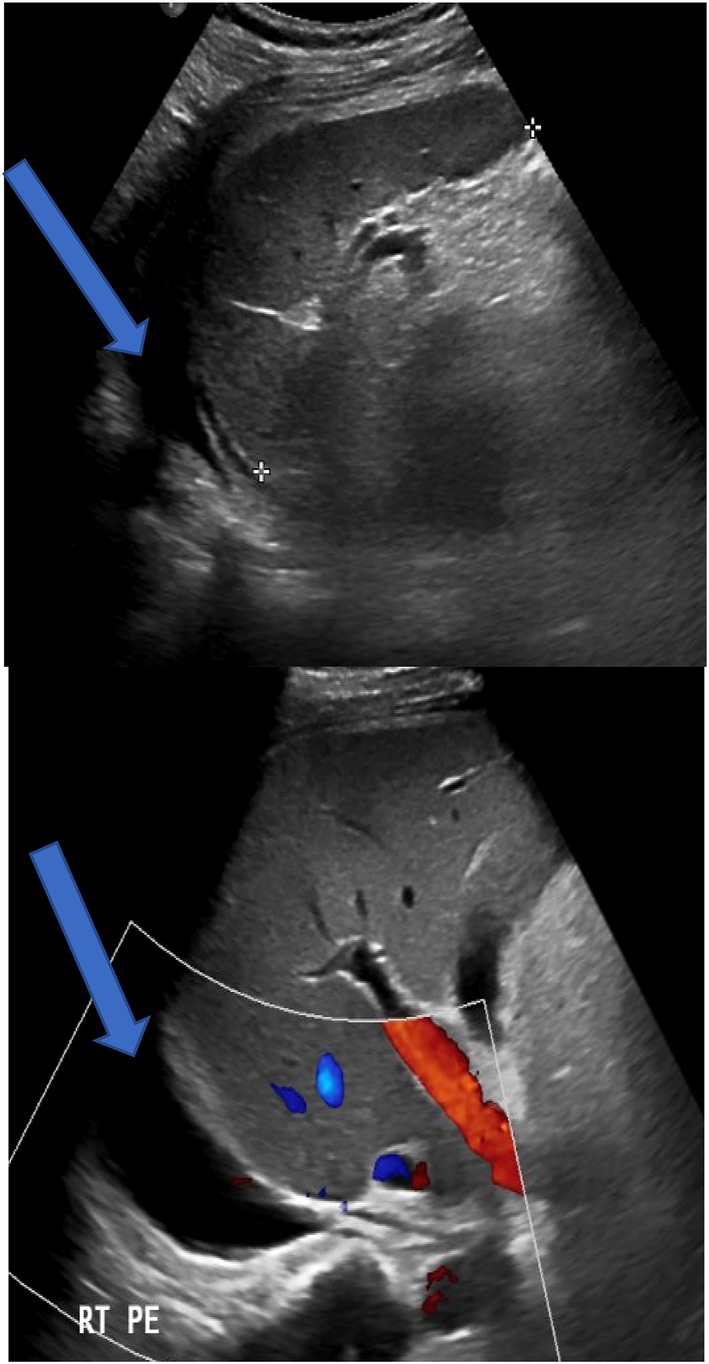
Doppler of liver: Fatty liver; patent hepatic vasculature; mild residual ascites (blue arrows).

Doppler of liver (04/23/2025) done after therapeutic ascites tapping: Fatty liver; patent hepatic vasculature; mild residual ascites and right pleural effusions (Figure 2).

**FIGURE 3:**
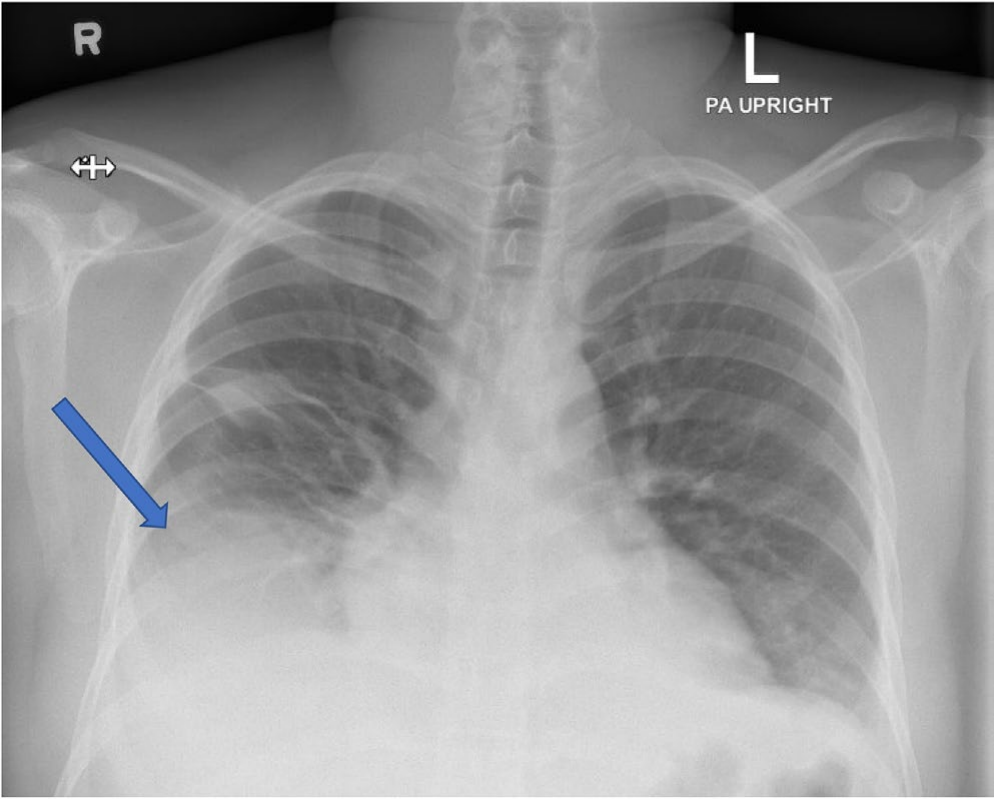
CXR: Right lower lobe consolidation with suspected pleural effusion (Blow arrow).

CXR: Right lower lobe consolidation with suspected pleural effusion (Figure 3).

**FIGURE 4:**
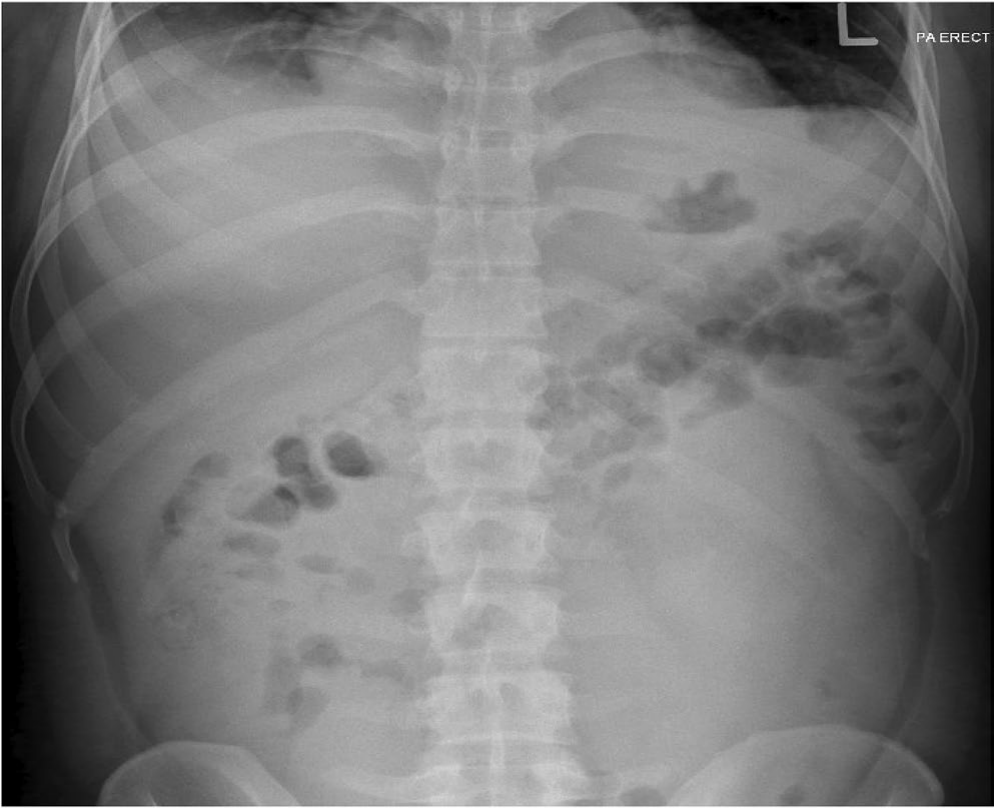
Abdomen X‐ray: No obstruction or perforation.

Abdomen X‐ray: No obstruction or perforation (Figure 4).

### 4.3 Ascitic Fluid Analysis

AFB smear and TB PCR‐positive, confirming tuberculous peritonitis.

### 4.4 Hospital Course

Initial management included hypertonic saline for hyponatremia and empiric antibiotics for pneumonia. After readmission with worsening ascites, paracentesis diagnosed TB peritonitis. ATT (rifampin, isoniazid, pyrazinamide, ethambutol) was initiated, with close monitoring for hepatotoxicity. Diuretics were avoided due to hypoalbuminemia; large‐volume paracentesis with albumin infusion was preferred. Gastrointestinal endoscopy was not pursued given the absence of any gastrointestinal symptoms.

### 4.5 Final Diagnoses

- Principal: Tuberculous peritonitis, right lower lobe pneumonia, heavy proteinuria, mild pleural effusion.
- Additional: Hypertension (HTN).

## 5 Discussion

Extrapulmonary TB (EPTB) accounts for 15%–20% of TB cases in immunocompetent individuals, with peritoneal involvement occurring in approximately 2% of cases [1]. This case illustrates the diagnostic challenges posed by EPTB, as the patient's lack of pulmonary symptoms and initial attribution of ascites to hypoalbuminemia from nephrotic‐range proteinuria delayed suspicion of TB [1, 4]. The clinical presentation underscores that EPTB can mimic malignancy or inflammatory disorders [5], emphasizing that in endemic regions, TB should remain a diagnostic consideration even in the absence of classic symptoms. Early diagnostic paracentesis proved critical in this case, as while AFB smear sensitivity remains low (≤ 10%), TB PCR significantly improves detection rates to approximately 80% [2], with PCR ultimately providing the pivotal diagnostic confirmation. Although a contrast‐enhanced CT of the chest and abdomen could potentially identify micronodules or lymphadenopathy suggestive of TB [6], it was not performed in this case as the diagnosis was already confirmed by ascitic fluid PCR and the patient demonstrated a rapid clinical response to ATT, thereby obviating the need for further radiation exposure and cost.

The patient's severe hyponatremia (Na 117 mEq/L) demonstrated the complex pathophysiology of TB, with multiple contributing factors requiring careful evaluation. Syndrome of inappropriate antidiuretic hormone secretion (SIADH) emerged as the most likely primary cause, driven by TB‐related inflammation stimulating ADH secretion [3]. Although hypovolemic hyponatremia secondary to ascites‐induced intravascular depletion was considered, the patient's euvollemic status ultimately favored SIADH as the predominant mechanism. Adrenal insufficiency was effectively ruled out by normal potassium and cortisol levels [7]. Management required meticulous attention to correction rates, utilizing 2% hypertonic saline and fluid restriction to prevent central pontine myelinolysis [8], with serial sodium monitoring every 6 h being absolutely imperative for safe management.

Concurrent acute kidney injury (AKI, creatinine 146 μmol/L) with heavy proteinuria presented additional diagnostic complexity, with several potential etiologies requiring differentiation. Tubulointerstitial nephritis (TIN) represented the leading hypothesis, given TB's known capacity to directly infiltrate renal tissue or trigger immune‐mediated injury [9]. Postinfectious glomerulonephritis was considered less likely due to the absence of hematuria or recent streptococcal infection [10], while obstructive uropathy was excluded by renal ultrasound demonstrating no hydronephrosis [11]. Although renal biopsy could have provided definitive etiologic clarification, the decision was made to defer this invasive procedure given the patient's clinical improvement following initiation of antitubercular therapy (ATT) [12].

Review of comparative literature reveals concerning patterns in TB peritonitis diagnosis, with studies reporting median diagnostic delays of up to 8 weeks, primarily attributable to nonspecific presentations [13]. While our patient presented with the unusual triad of AKI, severe hyponatremia, and ascites, Daher et al. reported a case series where AKI in TB peritonitis was often multifactorial, though rarely the presenting feature [13]. In contrast to many case series that describe patients with evident pulmonary involvement or known risk factors [14], this case is distinctive for the complete absence of pulmonary symptoms and the initial diagnostic focus on primary renal pathology, underscoring the broad and unpredictable spectrum of EPTB. The diagnostic challenge of abdominal TB is well documented, often requiring a high index of suspicion as it can mimic various other conditions [15]. Furthermore, tuberculous peritonitis can co‐exist with intestinal tuberculosis even in the absence of gastrointestinal symptoms, underscoring the importance of considering endoscopic evaluation in selected cases where the diagnosis remains uncertain or there is a poor response to therapy [16]. The nonspecific nature of symptoms frequently leads to diagnostic delays, reinforcing the value of molecular diagnostic techniques like PCR on ascitic fluid [17].

Imaging modalities including ultrasound, while potentially revealing suggestive findings like thickened peritoneum or lymphadenopathy [6], frequently lack specificity, as demonstrated in this case where imaging primarily highlighted ascites but required definitive fluid analysis for diagnostic confirmation. Therapeutic management presented its own challenges, particularly the need for vigilant LFT monitoring due to rifampin's hepatotoxic potential [18], while hypoalbuminemia guided the approach to ascites management, favoring paracentesis over diuretics [19].

From a public health perspective, this case highlights important opportunities for improving diagnostic pathways in TB‐endemic regions, particularly through implementing protocols for early ascitic fluid PCR in cases of unexplained ascites. The successful management of this complex presentation ultimately depended on effective multidisciplinary collaboration, with coordinated efforts between nephrology, infectious disease, and radiology specialists proving critical for timely diagnosis and appropriate ATT initiation. This approach not only addressed the immediate clinical challenges but also optimized long‐term patient outcomes.

## 6 Conclusion

This case highlights the requirement for a high index of suspicion in unusual presentations and the diagnostic difficulties of extrapulmonary tuberculosis (EPTB) posing as acute kidney injury (AKI) and hyponatremia. Even in the absence of pulmonary symptoms, tuberculous peritonitis should be taken into consideration in all cases of unexplained ascites since early detection has a major impact on results. Because of its higher sensitivity, PCR‐based ascitic fluid analysis should be prioritized over traditional smear microscopy in order to improve diagnostic accuracy. For healthcare systems in TB‐endemic regions, implementing a simple diagnostic protocol that includes early ascitic fluid TB‐PCR analysis for all cases of unexplained lymphocytic exudative ascites could significantly reduce diagnostic delays, minimize unnecessary investigations, and facilitate earlier initiation of treatment. To effectively manage concurrent hyponatremia, AKI, and the start of antitubercular medication while minimizing potential consequences, a multidisciplinary approach is necessary. To improve recovery and management tactics, more investigation into the long‐term renal consequences of TB‐associated AKI is also necessary.

## Author Contributions

**Bassem Al Hariri:** supervision, writing – original draft, writing – review and editing. **Muhammad Sharif:** validation, writing – original draft, writing – review and editing. **Memon Noor Illahi:** supervision, writing – original draft, writing – review and editing. **Asgad Mohamed Ahmed Eltigani:** writing – original draft, writing – review and editing. **Usamah Saad Mohammad Al Anbagi:** validation, writing – original draft, writing – review and editing. **Muayad Ahmad:** supervision, validation, writing – review and editing.

## Funding

The authors have nothing to report.

## Ethics Statement

This patient provided oral and signed written consent to use his clinical materials in this study. The study was conducted by the principles of the institutional ethical standards and national research committee.

## Consent

Written informed consent was obtained from the patient for publication of this case report and any accompanying images.

## Conflicts of Interest

The authors declare no conflicts of interest.

## Data Availability

The data that support the findings of this study are available from the corresponding author upon reasonable request.
